# Subcutaneous fat mass is associated with genetic risk scores related to proinflammatory cytokine signaling and interact with physical activity in middle-aged obese adults

**DOI:** 10.1186/s12986-019-0405-0

**Published:** 2019-11-08

**Authors:** James W. Daily, Hye Jeong Yang, Meiling Liu, Min Jung Kim, Sunmin Park

**Affiliations:** 1 Department of R&D, Daily Manufacturing Inc., Rockwell, NC 28138 USA; 2 Food Functional Research Division, Korean Food Research Institutes, Sungnam, 55365 South Korea; 3 Department of Food and Nutrition, Obesity/Diabetes Research Center, Hoseo University, Asan, Chungnam 31499 South Korea; 4 Food and Nutrition, Hoseo University, 165 Sechul-Ri, BaeBang-Yup, Asan-Si, ChungNam-Do 336-795 South Korea

**Keywords:** Subcutaneous fat, Visceral fat, Waist circumferences, Lipid metabolism, Glucose metabolism

## Abstract

**Background and aims:**

Subcutaneous fat mass is negatively correlated with atherogenic risk factors, but its putative benefits remain controversial. We hypothesized that genetic variants that influence subcutaneous fat mass would modulate lipid and glucose metabolism and have interactions with lifestyles in Korean middle-aged adults with high visceral fat.

**Materials and methods:**

Subcutaneous fat mass was categorized by dividing the average of subscapular skin-fold thickness by BMI and its cutoff point was 1.2. Waist circumferences were used for representing visceral fat mass with Asian cutoff points. GWAS of subjects aged 40–65 years with high visceral fat (*n* = 3303) were conducted and the best gene-gene interactions from the genetic variants related to subcutaneous fat were selected and explored using the generalized multifactor dimensionality reduction. Genetic risk scores (GRS) were calculated by weighted GRS that was divided into low, medium and high groups.

**Results:**

Subjects with high subcutaneous fat did not have dyslipidemia compared with those with low subcutaneous fat, although both subject groups had similar amounts of total fat. The best model to influence subcutaneous fat included *IL17A*_rs4711998, *ADCY2_*rs326149, *ESRRG_*rs4846514, *CYFIP2_*rs733730, *TCF7L2_*rs7917983, *ZNF766_*rs41497444 and *TGFBR3*_rs7526590. The odds ratio (OR) for increasing subcutaneous fat was higher by 2.232 folds in the high-GRS group, after adjusting for covariates. However, total and LDL cholesterol, triglyceride and C-reactive protein concentrations in the circulation were not associated with GRS. Subjects with high-GRS had higher serum HDL cholesterol levels than those with low-GRS. Physical activity and GRS had an interaction with subcutaneous fat. In subjects with low physical activity, the odds ratio for high subcutaneous fat increased by 2.232, but subcutaneous fat deposition was not affected in the high-GRS group with high physical activity.

**Conclusion:**

Obese adults with high-GRS had more subcutaneous fat, but they did not show more dyslipidemia and inflammation compared to low-GRS. High physical activity prevented subcutaneous fat deposition in subjects with high GRS for subcutaneous fat.

## Introduction

Obesity is increasing worldwide. In Korea, obesity is defined as > 25% fat for men and > 30% fat for women [1]. However, not only fat mass itself but also fat distribution is associated with metabolic diseases. In people with similar total fat masses, subcutaneous fat mass is negatively correlated with atherogenic metabolic risk factors [2] and visceral fat deposition initiates a dysfunctional state in the insulin-sensitive tissues leading to insulin resistance [3]. Visceral adipose tissues, which contain high numbers of macrophages, produce proinflammatory cytokines including tumor necrosis factor-α (*TNF- α*) and interleukin-6 (*IL-6*) and less adiponectin. High circulating free fatty acid levels by lipolysis also increases insulin resistance by stimulating very low-density lipoprotein (VLDL) production in the liver. VLDL transfers triglyceride to high-density lipoprotein (HDL) to make triglyceride rich HDL that are subsequently hydrolyzed by hepatic lipase, leading to small HDL. The increase of serum free fatty acid and proinflammatory cytokine concentrations, mainly from visceral fat, leads to insulin resistance which promotes metabolic diseases [4]. However, a recent study [5] has demonstrated that subcutaneous fat tissue is the largest fat depot in the body, and its lipolysis may influence the increase serum free fatty acid levels. Consequently, a high lipolysis rate from subcutaneous fat depot may induce hypertriglyceridemia and low HDL and subcutaneous fat tissue may lead to dyslipidemia [5]. Thus, it is still uncertain whether the subcutaneous fat depot influences the development of metabolic diseases.

There are genetic differences between visceral fat and subcutaneous fat [2]. Genes with fat depot-specific expression in subcutaneous vs visceral adipose tissue provide candidate genes for involvement in the regulation of fat distribution. Genome wide association study (GWAS) identified several polymorphisms in developmental genes including T-box 15 (*TBX15*), homeobox C13 (*HOXC13*), R-spondin 3 (*RSPO3*) and cytoplasmic polyadenylation element binding protein 4 (*CPEB4*) that are strongly associated with fat distribution [6]. Adamska-Patruno et al. have demonstrated that certain single-nucleotide polymorphisms (SNP) of melanocortin-4-receptor (*MC4R*) gene have a significant association with visceral fat and alteration of postprandial carbohydrate utilization in Europeans [7]. However, rs17782313 of MC4R has a significant association with general obesity as determined by body mass index (BMI) in Koreans and had an interaction with mental stress and energy intake [8]. Wang et al. [9] have also reported that only in Chinese women, rs17782313 near *MC4R* and rs4846567 near lysophospholipase like 1 (*LYPLAL1*) are associated with visceral fat mass and subcutaneous fat mass, respectively (*P* = 2.93 × 10^− 4^ and 0.0015, respectively). Only in men, rs671 in aldehyde dehydrogenase 2 family member (*ALDH2*) is associated with visceral fat mass (*P* = 1.75 × 10^− 8^). These results suggest that fat distribution is influenced by genetic variants and they have interactions with environmental factors.

Overall obesity and central obesity are commonly defined by BMI, waist circumferences, waist-and-hip ratio, and body fat measured by Bioelectrical Impedance Analysis, although they are not precise measurements of fat distribution. Skin-fold measurement is a well-established method to measure subcutaneous fat mass [5]. However, fat distribution measured by magnetic resonance imaging (MRI) is more precise than waist circumference and skin-fold measurement for distinguishing between visceral fat and subcutaneous fat. However, MRI is difficult to use for adiposity measurements in large scale studies. Skin-fold thickness measurement by a caliper is used for determining subcutaneous fat in biceps skinfold (front side middle upper arm), triceps skinfold (back side middle upper arm), subscapular skinfold (under the lowest point of the shoulder blade), suprailiac skinfold (above the upper bone of the hip). Thus, persons can be categorized according to distribution of visceral and subcutaneous fat mass by the skin-fold thickness measurement and waist circumferences. The present study hypothesized that genetic variants that influence subcutaneous fat mass would have a combined effect that would constitute a genetic risk score that would indicate a person’s likelihood of depositing fat subcutaneously. The genetic trait for preferably storing excess energy as subcutaneous fat would be expected to modulate lipid and glucose metabolism and to have interactions with lifestyles. In persons with low visceral fat, subcutaneous fat influences the obesity and the genetic variants selected are difficult to be distinguished from obesity related genetic variants since different total fat contents were different between the person with low visceral fat and high subcutaneous fat and those with low visceral fat and low subcutaneous fat. The hypothesis was tested in Korean adults with high visceral fat with low and high subcutaneous fat in the Ansan/Ansung cohort of the Korean Genome and Epidemiology Study (KoGES).

## Methods

### Subjects in Ansan/Ansung cohorts

Adults aged 40–69 years (*n* = 10,004) who resided in the Ansan (city community) or Ansung (rural community) cohorts for over 6 months were included in the KoGES. There were 10,004 participants selected for the large-scale genome-wide genotyping and genetic variants for 8842 adults were released for research.

### Anthropometric measurement and experimental grouping for subcutaneous and visceral obesity

Anthropometric data including height, weight, and circumferences of waist and hip were measured in subjects wearing light clothes with bare feet [10]. BMI was calculated by dividing the body weight in kilograms by the height in meters squared. Body fat was estimated by the tetrapolar bioelectrical impedance method with an Inbody 3.0 (Biospace Co., Ltd., Seoul, Korea). Skin-fold thickness at subscapular and suprailiac sites was measured in triplicate with bare skin using a caliper with a dial graduation of 0.2 mm by skilled technicians [11]. Participants had an upright posture with relaxation. The subscapular skinfold was determined just below the right scapula with the fold pinched at an angle of 45° to the spine. The suprailiac skinfold was measured in the site in line with the natural angle of the iliac crest at the anterior axillary line. Skinfold measurement was successively conducted three times and the average of the three values was used [11]. Since skin-fold thickness at the suprailiac site was difficult to distinguish from visceral fat mass, that at the subscapular site was used to represent subcutaneous fat mass. Subcutaneous fat mass was calculated by dividing the average of subscapular skin-fold thickness by BMI. The cutoff-point of subcutaneous fat mass was 1.2 cm [11, 12]. Waist circumferences were used for representing visceral fat mass since waist circumference is a reliable index for visceral adiposity [13, 14]. The cutoff points of waist circumferences were 90 cm for men and 80 cm for women, according to Asian cutoff points. Subjects were divided into 4 groups according to skin-fold thickness at the subscapular site and waist circumferences as follows: 1) high subcutaneous fat and low visceral fat (HS-LV), 2) low subcutaneous fat and low visceral fat (LS-LV), 3) high subcutaneous fat and high visceral fat (HS-HV) and 4) low subcutaneous fat and high visceral fat (LS-HV). Since obesity is associated with visceral and subcutaneous fat, subjects with low visceral fat were eliminated. HS-HV (case) and LS-HV (control) were used for investigating genetic impact for subcutaneous fat.

### Lifestyles

Socioeconomic information of the subjects was collected during a health interview. Education level was categorized into less than high school, high school, and college or more. Smoking status was divided into current smoker, past smoker, and never-smoker. Daily alcohol consumption was calculated by multiplying alcohol contents by drinking amount per day. Alcohol intake status was categorized into four groups according to average daily alcohol consumption: nondrinker, light drinker (1–15 g), moderate drinker (16–30 g), and heavy drinker (> 30 g) (Table 1). Coffee intake was estimated by the frequencies of drinking one or more serving size of coffee per day and the subjects were divided into 3 groups including none (< 3 cup), moderate (3–10 cups/week) and heavy (> 10 cups/week).

**Table 1:** The characteristics of subjects according to subcutaneous and visceral fat mass

	HS-LV (*n* = 1190)	LS-LV (*n* = 4349)	HS-HV (*n* = 1016)	LS-HV (*n* = 2287)
Age (years)	49.6 ± 7.2^b^	49.9 ± 8.9^b^	54.4 ± 8.5^a^	54.3 ± 4.7^a+++^
Gender (male, %)	456(38.3)	2924 (67.2)	208 (20.5)	595 (26.0)^***^
Residence area (Rural %)	94.5	52.3	67.8	23.7^***^
Obesity (≥25 kg/m^2^)	475 (39.9)	718 (16.5)	737 (72.5)	1132 (49.5)^***^
Physical Activity (%)				
Little (< 10 min/day)	62.6	44.8	59.6	40.7^***^
Moderate (10–60 min/day)	33.8	34.3	28.1	31.9
Many (≥60 min/day)	3.58	20.9	12.3	27.5
Alcohol intake (%)				
None to Little (< 20 g/day)	87.5	78.2	89.9	90.2^***^
Moderate to heavy (≥20 g/day)	12.5	21.8	10.1	9.79
Coffee intake (%)				
Little (< 1 cup/week)	19.5	23.9	23.4	31.5^***^
Moderate (1–10 cups/week)	77.5	70.8	71.8	63.1
Heavy (> 10 cups/week)	3.0	5.3	4.8	5.4
Smoking intake (%)				
No smoking	69.5	42.6	79.7	75.4^***^
Past smoking	13.4	20.6	8.74	10.0
Smoking	17.1	36.8	11.5	14.6
Energy intake (Kcal/day)	1878 ± 594^b^	1888 ± 605^ab^	1918 ± 694^ab^	1966 ± 858^a+^
Carbohydrate intake (En%)	70.5 ± 5.7^b^	70.8 ± 7.0^ab^	70.2 ± 7.1^b^	71.2 ± 7.1^a**^
Protein intake (En%)	13.8 ± 4.4^ab^	13.6 ± 5.4^b^	14.0 ± 5.5^a^	13.6 ± 5.5^b***^
Fat intake (En%)	14.8 ± 2.0^ab^	14.6 ± 2.4^ab^	15.1 ± 2.5^a^	14.5 ± 2.5^b**^

Subjects were categorized into 4 groups according to subcutaneous fat (cutoff point: 1.2 of the ratio of subcutaneous fat in subscapular site and BMI) and visceral fat (cutoff point: men, 90 cm and women, 80 cm of waist circumferences). *HS-LV* high subcutaneous fat and low visceral fat, *LS-LV* low subcutaneous fat and low visceral fat, *HS-HV* high subcutaneous fat and high visceral fat, *LS-HV* low subcutaneous fat and high visceral fat

^*^^*^Significant differences by subcutaneous fat in subscapular site by two-way ANOVA at *P* < 0.01, ^***^ *P* < 0.001

^+^Significant difference by visceral fat by waist circumference by two-way ANOVA at *P* < 0.05, ^+++^ *P* < 0.001

^a,b,c^Means without a common letter differ in the same row at *P* < 0.05

Regular physical activity was determined by multiplying the duration by intensity of exercise and the subjects were categorized into 3 groups including little (< 10 min/day), moderate (10–30 min/day) and heavy (> 30 min/day) activity [8]. Mental stress was evaluated by asking subjects to 10 questions concerning their state of agitation and anxiety in the workplace and family situations in their daily life as described in the previous study [8]. The severity of mental stress was calculated by the sum of all answers. Mental stress was categorized into three groups such as mild stress (< 2), moderate stress (2–5) and severe stress (≥6).

### Laboratory biochemical tests

Blood samples were obtained after 12 h or more fasting. HbA1c from whole blood, plasma glucose concentrations, and serum concentrations of total and HDL-cholesterol and triglycerides were measured using an automatic analyzer (ZEUS 9.9; Takeda, Tokyo, Japan). Serum LDL cholesterol concentrations were calculated by the Freidman equation. Serum insulin concentrations were measured using a gamma counter (1470 Wizard; Perkin-Elmer, San Jose, CA, USA) with a radioimmunoassay kit (DiaSorin, Stillwater, MN, USA). Homeostasis model assessment estimates (HOMA) were used to estimate beta (B) cell capacity (HOMA-B) and insulin resistance (HOMA-IR). HOMA assessments were calculated as previously described [15]. Systolic (SBP) and diastolic blood pressure (DBP) were measured three times on the right arm at the same height as their heart in a sitting position with a 1 min interval between measurements; we used the averages of the three measurements of SBP and DBP. Serum C-reactive protein (CRP) was measured by ELISA kit.

### Assessment of food and nutrient intake by semi-quantitative food frequency questionnaires (SQFFQ)

Dietary intake was estimated using a SQFFQ that was developed and validated for the KoGES. This questionnaires requested information regarding the participants’ consumption of food items. The SQFFQ was developed and validated for the KoGES and it included 103 food items [16]. An average daily nutrient intake was calculated from the food intake measured by SQFFQ using the Computer-Aided Nutritional Analysis Program (CAN Pro) 3.0, which is a nutrient database developed by the Korean Nutrition Society.

### Genotyping and quality control

The genetic variants of 8842 subjects were given by the Center for Genome Science, the Korea National Institute of Health. Genomic DNA was extracted from whole blood and genotypes measured on an Affymetrix Genome-Wide Human SNP Array 5.0 with 500,568 SNPs (Affymetrix, Santa Clara, CA). The genotype accuracy was checked by internal sample quality control process including Bayesian Robust Linear Modeling using the Mahalanobis Distance Genotyping Algorithm [17]. The exclusion criteria of genotypes were as follows: high heterozygosity (> 30%), high missing genotype call rates (≥4%), gender biases or Hardy-Weinberg equilibrium test (*P* < 0.05). More than 10% of missing genotypes were excluded from the analysis and 440,794 SNPs were included in the GWAS. The reproducibility and validity of the SNPs were conducted by the Korean Center for Disease and Prevention (Osong, Korea).

### The best model for gene-gene interaction by GMDR

GWAS was conducted in HS-HV (Case) and LS-HV (Control). We selected 90 SNPs of the genes related to inflammation and estrogen signaling to be associated with high subcutaneous fat thickness from the SNPs selected from the GWAS (*P* < 0.0001). The significant genetic variants linked to the high subcutaneous fat thickness in subjects with high waist circumferences were identified by GWAS using GPLINK (*P* < 0.05). The genes related to pro-inflammatory cytokines and their signaling were selected and the genetic variants of the selected genes were included from the GWAS (*P* < 0.05). The generalized multifactor dimensionality reduction (GMDR) method was used to select the genetic variants to have gene-gene interactions [10, 18]. GMDR was a nonparametric and genetic model for detecting and characterizing nonlinear interactions among discrete genetic attributes. We found gene-gene interaction models to be related to high subcutaneous fat mass in the GMDR models. The best model of gene-gene interaction was found based on trained balance accuracy (TRBA), test balance accuracy (TEBA) and cross-validation consistency (CVC) in the GMDR models [19].

Genetic risk scores (GRS) for the best model was calculated for each subject by summing the number of risk alleles from each selected SNP in the best model. The GRS was divided into 3 categories by its tertiles (0–7, 8–9, and 10–12). The GRS indicated that the subjects with high-GRS were at higher risk for high subcutaneous fat than those with low-GRS.

### Statistical analysis

Statistical analysis was performed using GPLINK version 2.0 (http://pngu.mgh.harvard.edu/ ~purcell/plink) and SAS (version 9.3; SAS Institute, Cary, NC, USA). The statistical differences of baseline characteristics for 4 groups (HS-LV, LS-LV, HS-HV, and LS-HV) were examined by two-way analysis of variance (ANOVA). Frequency distributions of categorical variables including gender, education levels, smoking status, etc. were assessed using the chi-squared test.

Since HS-HV and LS-HV had similar levels of total body fat with higher subcutaneous fat in HS-HV and with lower subcutaneous fat in LS-HV, the genetic variants that affect subcutaneous fat mass were selected by comparing HS-HV (case) and LS-HV (control) by GWAS. After conducting GWAS, the genetic variants that met the HWE (*P* > 0.05) and not highly conservative (linkage disequilibrium, *P* < 0.05) were included in GMDR to find the best model with the genetic variants to influence subcutaneous fat mass. Major allele, heterozygote allele, and minor allele of each selected SNP in the best model were assigned to 0, 1, and 2, respectively. GRS was calculated by summing the assigned value of each allele multiplied by coefficient value of logistic regression analysis in the best model of GMDR. GRS was categorized into three groups by the tertiles of GRS: low, medium, and high (0–6, 7–8, and > 8). Two-way analysis of variance (ANOVA) was conducted to explore the statistical differences by subcutaneous fat and GRS categories in each continuous variable such as age, subcutaneous fat, serum levels of total cholesterol, HDL cholesterol, LDL cholesterol, triglyceride, and glucose, HOMA-IR, and HOMA-B.

The association of GRS with the risk of subcutaneous fat and parameters related to subcutaneous fat was examined using logistic regression analysis, after adjustment for two different sets of covariates as model 1 and model 2. Model 1 included residence area, sex, age and BMI as covariates. Model 2 included model 1 plus smoking status, drinking status, coffee intake, physical activity, energy intake and fat intake as energy percent. The odds ratios (ORs) and 95% confidence intervals (CI) were calculated based on Low-GRS as a reference.

To determine the interaction between the GRS and lifestyles, including dietary intake, a multivariate general linear model (GLM) analysis with interaction was conducted to evaluate the main effects of subcutaneous fat mass and its interaction effect with lifestyles after adjustment for covariates. As there was an interaction in the multivariate GLM, logistic regression analysis was performed in two groups using the assigned cutoff in each parameter of lifestyles. The cutoffs of each parameter were provided in the table legend. Subjects were categorized into higher and lower intake groups with the classification criterion. *P*-value ≤0.05 was considered statistically significant.

## Results

### Cohort characteristics

Characteristics of the subjects and nutrient intakes were summarized according to the subcutaneous and visceral fat mass (Table 1). HS-LV and LS-LV groups were younger than the HS-HV and LS-HV groups (*P* < 0.001), indicating that subjects with high visceral fat mass were older than those with low visceral fat regardless of subcutaneous fat mass. Age was a good determinant for visceral fat mass, since visceral fat mass increased with increasing age (*P* < 0.001; Table 1). More males were in the low visceral and low subcutaneous fat group (LS-LV) and more females were in the low visceral and high subcutaneous fat group (HS-HV, *P* < 0.0001; Table 1). About 34.6% of subjects were obese and HS-HV (72.5%) and LS-HV (49.5%) included obese subjects more than other groups (*P* < 0.001; Table 1). Physical activity, alcohol intake, coffee intake, smoking status and stress levels had significant differences among the different subcutaneous and visceral fat groups (*P* < 0.001; Table 1). Energy intake influenced visceral fat mass and it was higher in LS-HV group than HS-LV and LS-LV groups (*P* < 0.05). However, composition of carbohydrate, protein and fat intake (En%) affected subcutaneous fat but not visceral fat (Table 1). Subjects with high subcutaneous fat mass had higher intakes of fat (*P* < 0.01) and protein (*P* < 0.001) than those with low subcutaneous fat, whereas subjects with high carbohydrate intake had higher visceral fat and lower subcutaneous fat (Table 1).

Visceral fat was associated with a greater BMI and total fat mass, was influenced by both subcutaneous fat and visceral fat mass (Table 2). Subjects with high visceral and subcutaneous fat mass (HS-HV) had the highest BMI but total body fat mass as measured by In Body equipment was not significantly different between HS-HV and LS-HV (Table 2). Subcutaneous fat mass in subscapular sites was significantly affected by subcutaneous fat mass (*P* < 0.001) but subcutaneous fat mass in the suprailiac site were significantly influenced by not only subcutaneous fat mass (*P* < 0.001) but also visceral fat (*P* < 0.001; Table 2). Lipid profiles including total, LDL, and HDL cholesterol and triglyceride concentrations were significantly influenced by visceral fat mass (*P* < 0.05), but not subcutaneous fat mass. Serum concentrations of total and LDL cholesterol were lower in LS-LV than the other groups and serum HDL concentrations were higher in LS-LV (*P* < 0.05; Table 2). Serum triglyceride concentrations were higher in the ascending order of HS-HV, LS-HV = HS-LV and LS-LV (*P* < 0.01). DBP and SBP were affected by subcutaneous fat mass. Fasting serum glucose and HbA1c levels were also influenced only by visceral fat mass (*P* < 0.01; Table 2). Furthermore, serum insulin levels and HOMA-IR were influenced only by visceral fat (*P* < 0.01) but HOMA-B was not significantly different by subcutaneous and visceral fat mass. Blood pressure was significantly affected only by visceral fat (*P* < 0.05) and SBP and DBP were not significantly different between HS-HV and LS-HV (Table 2). Serum CRP concentrations, an index of inflammation, tended to be higher in subjects with high visceral fat mass (HS-HV and LS-HV) than those with low visceral fat mass (HS-LV and LS-LV) (*P* < 0.05). However, they tended to be lower with high subcutaneous fat; the only significant difference CRP in HV-LS was significantly higher than HS-LV suggesting that the combination of high visceral fat with low subcutaneous fat was the most pro-inflammatory combination. It suggested that subcutaneous fat might protect against inflammation. (Table 2). Therefore, visceral fat mass, but not subcutaneous fat mass, had an association with impaired lipid and glucose metabolism, and subcutaneous fat might protect against inflammation.

**Table 2:** Adjusted means and standard deviations of anthropometric and metabolic parameters according to the subcutaneous and visceral fat masses^1^

	HS-LV (*n* = 1190)	LS-LV (*n* = 4349)	HS-HV (*n* = 1016)	LS-HV (*n* = 2287)
Body mass index (kg/m^2^)	24.3 ± 2.0^c^	22.8 ± 2.4^d^	28.1 ± 2.7^a^	27.0 ± 2.7^b***+++^
Body fat (%)	27.8 ± 5.1^a^	26.6 ± 5.8^b^	27.3 ± 5.2^a^	27.3 ± 5.5^a***^
Waist circumference (cm)	80.0 ± 6.3^b^	79.4 ± 6.7^b^	86.5 ± 6.8^a^	87.4 ± 6.1^a+++^
Subcutaneous fat in subscapular site (mm)	34.2 ± 5.8^b^	20.6 ± 6.2^c^	36.5 ± 6.4^a^	20.3 ± 5.9^c***^
Subcutaneous fat in suprailiac site (mm)	29.8 ± 11.5^a^	24.2 ± 9.0^c^	30.5 ± 14^a^	26.1 ± 9.4^b***++^
Serum total cholesterol (C) (mg/dl)	195.1 ± 35.5^a^	189.4 ± 35.1^b^	199.8 ± 37.8^a^	197.6 ± 35.5^a+^
Serum LDL-C (mg/dl)	118.3 ± 32.7^a^	113.6 ± 33.5^b^	119.8 ± 34.0^a^	118.9 ± 33.5^a+^
Serum HDL-C (mg/dl)	44.0 ± 9.7^b^	46.5 ± 10.6^a^	44.0 ± 9.1^b^	43.6 ± 9.2^b+^
Serum TG (mg/dl)	163.4 ± 87.9^b^	141.5 ± 103.6^c^	180.2 ± 115.8^a^	175.2 ± 109.7^a++^
DBP (mmHg)	74.1 ± 11.7^ab^	73.9 ± 11.3^b^	75.4 ± 11.7^a^	75.2 ± 11.1^a++^
SBP (mmHg)	115.7 ± 16.4^b^	115.6 ± 17.5^b^	117.9 ± 18.3^a^	117.0 ± 18.7^ab+^
HbA1c (%)	5.8 ± 0.9^b^	5.7 ± 0.9^c^	6.0 ± 1.1^a^	5.8 ± 1.0^b+++^
Plasma glucose (mg/dl)	88.3 ± 22.7^b^	86.5 ± 20.7^b^	92.1 ± 23.0^a^	89.8 ± 21.9^a++^
Serum insulin (μIU/mL)	7.05 ± 3.27^b^	6.51 ± 3.87^b^	9.46 ± 5.12^a^	8.88 ± 5.89^a+++^
HOMA-IR	1.63 ± 0.9^b^	1.55 ± 1.0^b^	1.83 ± 1.3^a^	1.78 ± 0.8^a+++^
HOMA-B	149.7 ± 124.2	148.5 ± 144.8	143.4 ± 135.2	152.2 ± 167.4
Serum CRP-1 (mg/dL)	0.21 ± 0.35^b^	0.23 ± 0.46^ab^	0.23 ± 0.28^ab^	0.27 ± 0.54^a*+^

Subjects were categorized into 4 groups according to subcutaneous fat (cutoff point: 1.2 of the ratio of subcutaneous fat in subscapular site and BMI) and visceral fat (cutoff point: men, 90 cm and women, 80 cm of waist circumferences). *HS-LV* high subcutaneous fat and low visceral fat, *LS-LV* low subcutaneous fat and low visceral fat, *HS-HV* high subcutaneous fat and high visceral fat, *LS-HV* low subcutaneous fat and high visceral fat, *TG* triglyceride, *SBP* Systolic blood pressure, *DBP* Diastolic blood pressure, *HbA1c* Hemoglobin A1c, *CRP* C-reactive protein, *HOMA* Homeostasis model assessment, *IR* insulin resistance, *B* insulin secretion

^1^Adjusted for age, gender, residence area, BMI, waist circumference, hip circumference, body fat, alcohol and coffee intake, physical activity, smoking status, and energy intake

^*^Significant differences by subcutaneous fat measured at the subscapular site in two-way ANOVA at *P* < 0.05, ^***^ *P* < 0.001

^+^Significant difference by visceral fat by estimated by waist circumference in two-way ANOVA at *P* < 0.05, ^++^ at *P* < 0.01, ^+++^ *P* < 0.001

^a,b,c^Means without a common letter differ in the same row at *P* < 0.05

Selection of the genetic variants associated with subcutaneous fat mass.

Since fat mass is known to be associated with inflammation and estrogen signaling, genes involved in inflammatory and estrogen signaling were selected for GMDR (Table 3). The final GMDR analysis included 10 SNPs such as interleukin 17A (*IL17A*) rs4711998, interleukin 5 receptor subunit alpha (*IL5RA*) rs2290610, *IL5RA* rs2290610, estrogen related receptor gamma (*ESRRG*) rs4846514, cytoplasmic FMR1 interacting protein 2 (*CYFIP2*) rs733730, transcription factor 7 like 2 (*TCF7L2*) rs7917983, zinc finger protein 766 (*ZNF766*) rs41497444, contactin 4 (*CNTN4)* rs17024684, transforming growth factor beta receptor 3 (*TGFBR3*) rs7526590 and adenylate cyclase 2 (*ADCY2*) rs326149 (Table 3). ORs of SNPs indicated that minor alleles of SNPs increased (OR > 1) or decreased (0 < OR < 1) subcutaneous fat mass. *ESRRG* rs4846514, *TGFBR3* rs7526590, *CNTN4* rs17024684, and *ADCY2* rs326149 had positive associations with subcutaneous fat mass but *IL5RA* rs2290610, rs2290610, *CYFIP2* rs733730, *TCF7L2* rs7917983, *IL17A* rs4711998 and *ZNF766* rs41497444 were negatively associated with it (Table 3). Interestingly, genes that were related to proinflammatory cytokines had a negative association with subcutaneous fat mass (Table 3).

**Table 3:** The characteristics of the ten genetic variants used for the generalized multifactor dimensionality reduction analysis

CHR^1^	SNP	Position^2^	Minor	Major	OR^3^	P_adjust^4^	MAF^5^	HWE^6^	GENE	Function
1	rs4846514	214,769,308	G	A	1.171	0.037	0.198	0.093	*ESRRG*	Intron variant
1	rs7526590	91,994,042	T	A	1.287	0.0002	0.291	0.122	*TGFBR3*	Intron variant
3	rs2290610	3,114,957	C	T	0.817	0.002	0.359	0.212	*IL5RA*	Missense variant
3	rs3792421	3,124,791	G	A	0.831	0.004	0.357	0.158	*IL5RA*	Intron variant
3	rs17024684	3,030,247	A	G	1.269	0.012	0.115	0.294	*CNTN4*	Intron variant
5	rs326149	7,865,445	G	T	1.283	0.0009	0.193	0.060	*ADCY2*	Intron variant
5	rs733730	156,665,645	T	C	0.862	0.034	0.260	0.782	*CYFIP2*	Intron variant
6	rs4711998	52,158,312	G	A	0.81 7	0.004	0.277	0.426	*IL17A*	Upstream transcript variant
10	rs7917983	114,722,872	T	C	0.792	0.002	0.213	0.281	*TCF7L2*	Upstream transcript variant
19	rs41497444	57,476,717	C	A	0.787	0.001	0.245	0.070	*ZNF766*	Upstream transcript variant

*ESRRG* estrogen related receptor gamma, *TGFBR3* transforming growth factor beta receptor 3, *IL5RA* interleukin 5 receptor subunit alpha, *CNTN4* contactin 4, *ADCY2* adenylate cyclase 2. *CYFIP2* cytoplasmic FMR1 interacting protein 2, *IL17A* interleukin 17A, *TCF7L2* transcription factor 7 like 2, *ZNF766* zinc finger protein 766

^1^The chromosome number of the gene SNP. ^2^The position of SNP in the chromosome. ^3^Odds ratio (OR) to influence subcutaneous fat thickness at subscapular site from GWAS analysis. ^4^*P* value for OR of the minor alleles of the SNP in GWAS analysis with adjusted for covariates of age, gender, residence area, and BMI. ^5^Minor allele frequency (MAF). ^6^*P* value for Hardy–Weinberg equilibrium (HWE)

### The best model for gene-gene interactions related to subcutaneous fat mass

The best model was selected by sign test for GMDR model and CVC. The best model included 7 SNPs: *IL17A* rs4711998, *IL5RA* rs2290610, *ESRRG* rs4846514, *CYFIP2* rs733730, *TCF7L2* rs7917983, *ZNF766* rs41497444 and *TGFBR3* rs7526590 (Table 4). This model exhibited that *P* = 0.001 for sign test and CVC = 10 (P = 0.001) with and without adjusting for age, sex, area, BMI. TRBA and TEBA of the best model was 0.7216 and 0.5605 in the model after adjusting for age, sex, area, and BMI (Table 4).

**Table 4:** Generalized multifactor dimensionality reduction (GMDR) analysis of factors that influence subcutaneous fat deposition at the subscapular site without and with adjusting for covariates

Model	No adjustment				Adjusted for age, sex, area, BMI			
	TRBA	TEBA	Sign test (*P* value)	CVC	TRBA	TEBA	Sign test (*P* value)	CVC
Model 1: TGFBR3 rs7526590	0.5367	0.5050	6(0.377)	6/10	0.5427	0.5017	7(0.172)	6/10
Model 1 plus IL17A rs4711998	0.5446	0.4964	4(0.828)	2/10	0.5543	0.5457	9(0.011)	9/10
Model 2 plus ADCY2 rs326149	0.5618	0.5063	5(0.623)	3/10	0.5720	0.5223	9(0.011)	5/10
Model 3 plus CYFIP2 rs733730	0.5797	0.5173	9(0.011)	2/10	0.5906	0.5216	8(0.055)	3/10
Model 4 plus ESRRG rs4846514	0.6094	0.5332	9(0.011)	7/10	0.6234	0.5248	7(0.172)	3/10
Model 5 plus TCF7L2 rs7917983	0.6500	0.5267	10(0.001)	6/10	0.6660	0.5302	8(0.055)	5/10
*Model 6 plus ZNF766 rs41497444*	0.7020	0.5321	10(0.001)	10/10	0.7216	0.5605	10(0.001)	10/10
Model 7 plus IL5RA rs2290610	0.7541	0.5213	8(0.055)	6/10	0.7707	0.5309	8(0.055)	10/10
Model 8 plus CNTN4 rs17024684	0.8008	0.4960	5(0.623)	6/10	0.8147	0.4978	4(0.828)	10/10
Model 9 plus IL5RA rs3792421	0.8343	0.4973	5(0.623)	10/10	0.8462	0.5058	6(0.377)	10/10

*TRBA* trained balanced accuracy, *TEBA* test balance accuracy, *CVC* cross-validation consistency; sign test, result and *P* value for the significance of GMDR model by sign test with and without adjusting for covariates; *BMI* body mass index, *ESRRG* estrogen related receptor gamma, *TGFBR3* transforming growth factor beta receptor 3, *IL5RA* interleukin 5 receptor subunit alpha, *CNTN4* contactin 4, *ADCY2* adenylate cyclase 2, *CYFIP2* cytoplasmic FMR1 interacting protein 2, *IL17A* interleukin 17A, *TCF7L2* transcription factor 7 like 2, *ZNF766* zinc finger protein 766

### Adjusted ORs for subcutaneous fat by GRS of the best model

Logistic regression analysis of parameters related to the subcutaneous fat mass and dyslipidemia were conducted after adjusting for residence area, gender, age, and BMI for model 1, and the confounding factors in model 1 plus, waist circumference, hip circumference, body fat, alcohol and coffee intake, physical activity, smoking status, and energy intake for model 2. Subjects in the high-GRS group had a higher subcutaneous fat mass by 2.317 and 2.232 folds in model 1 and model 2, respectively (*P* < 0.001; Table 5). Serum total and LDL cholesterol and triglyceride levels were not associated with GRS (Table 5). However, serum HDL cholesterol levels had a negative association with GRS: subjects with the high-GRS had a significantly lower serum HDL cholesterol than low-GRS only in model 1 (*P* < 0.01). Serum glucose levels were not associated with GRS (Table 5). Serum CRP concentrations were not significantly associated with GRS in model 1 and model 2. Therefore, subjects with high-GRS had higher subcutaneous fat but they had similar levels of serum CRP (Table 5).

**Table 5:** Adjusted odds ratios for metabolic disease risk factors according to the genetic risk scores (GRS) of model 7 for subcutaneous fat deposition

	Model 1			Model 2	
	Low-GRS (*n* = 999)	Medium-GRS (*n* = 1551)	High-GRS (*n* = 744)	Medium-GRS (*n* = 1551)	High-GRS (*n* = 744)
Subcutaneous fat (mm)	1	1.703 (1.385~2.093)^***^	2.317 (1.826~2.940)^***^	1.697 (1.334~2.159)^***^	2.232 (1.676~2.972)^***^
Waist circumference (cm)	1	0.990 (0.801~1.222)	1.046 (0.813~1.347)	1.128 (0.869~1.465)	1.105 (0.803~1.522)
Serum total cholesterol (mg/dl)	1	1.220 (0.981~1.517)	1.152 (0.889~1.494)	1.289 (0.998~1.665)	1.087 (0.795~1.488)
Serum HDL (mg/dl)	1	0.804 (0.683~0.948)^**^	0.709 (0.581~0.864)^**^	0.841 (0.688~1.029)	0.827 (0.646~1.057)
Serum LDL (mg/dl)	1	0.983 (0.790~1.224)	1.011 (0.780~1.312)	1.047 (0.807~1.359)	1.051 (0.768~1.438)
Serum TG (mg/dl)	1	0.878 (0.735~1.049)	0.986 (0.799~1.217)	0.960 (0.774~1.192)	1.036 (0.798~1.345)
Plasma glucose (mg/dL)	1	0.994 (0.676~1.463)	0.933 (0.584~1.490)	0.952 (0.615~1.473)	0.935 (0.549~1.595)
Serum CRP-1 (mg/dL)	1	1.321 (0.984~1.790)	1.218 (0.886~1.677)	1.384 (0.972~2.008)	1.440 (0.977~2.123)

*HDL* high density lipoprotein cholesterol, *LDL* low-density lipoprotein cholesterol, *TG* triglyceride, *CRP-1* C-reactive protein

Values represent odd ratios and 95% confidence intervals

GRS was divided into 3 categories by tertiles of GRS in the model which included *IL17A* rs4711998, *ADCY2* rs326149, *ESRRG* rs4846514, *CYFIP2* rs733730, *TCF7L2* rs7917983, *ZNF766* rs41497444 and *TGFBR3* rs7526590

Low-GRS was the reference for both model 1 and model 2

^**^Significantly different from low GRS in logistic regression analysis at *P* < 0.01, ^***^ *P* < 0.001

Model 1: Adjusted for age, gender, residence area, and BMI

Model 2: Adjusted for age, gender, residence area, BMI, waist circumference, hip circumference, body fat, alcohol and coffee intake, physical activity, smoking status, and energy intake

### Interaction of GRS and lifestyles including nutrient intakes

**Fig. 1:**
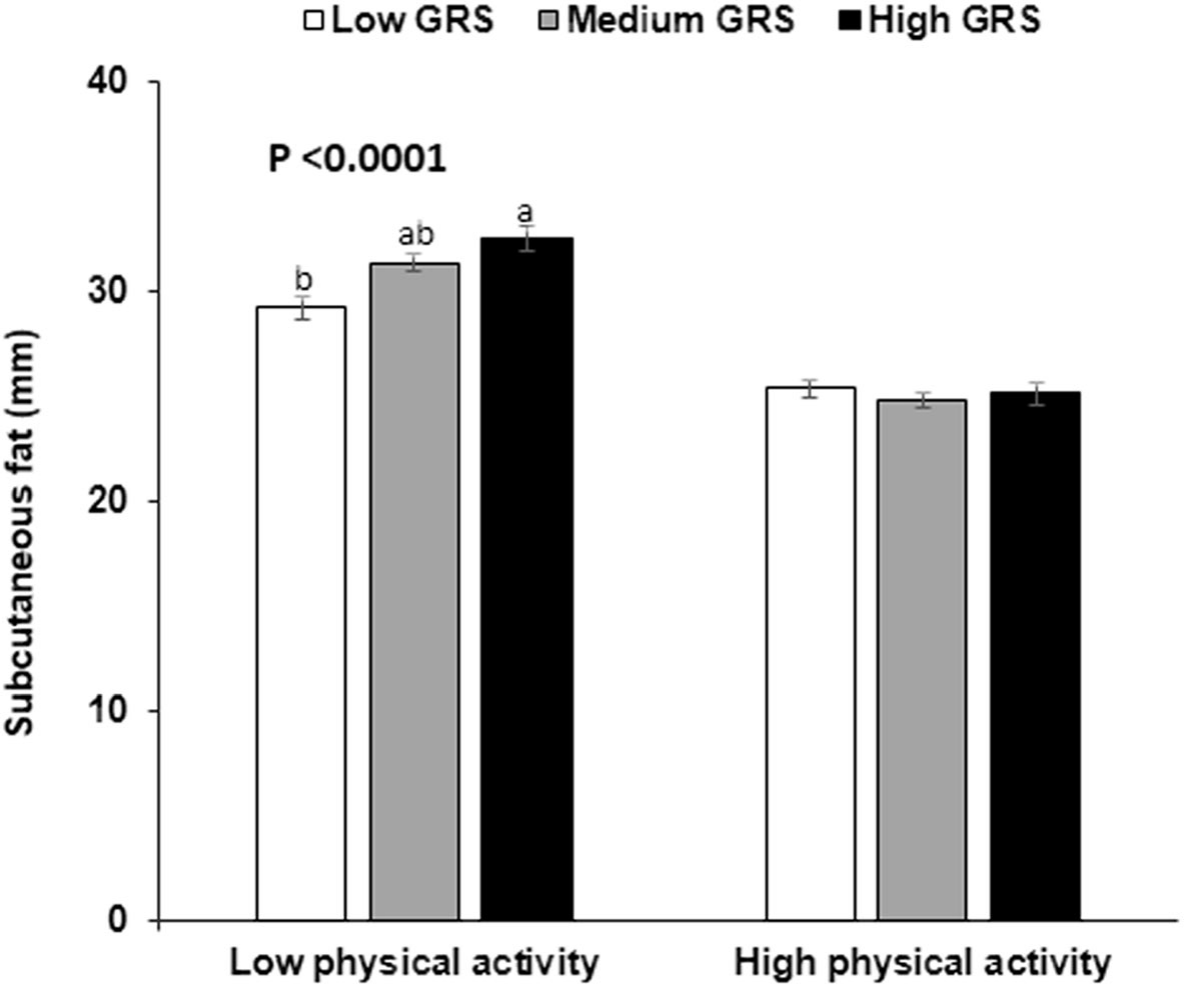
The amount of subcutaneous fat in the subjects according to the GRS alleles in high and low physical activity. The cutoff point of low and high physical activity was 1 h moderate activity per day. *P* values indicate the significance of subcutaneous fat amount according to GRS alleles in low physical activity. ^a,b^ Bars without a common letter differ at *P* < 0.05

GRS had no interaction with daily energy, carbohydrate, fat or protein intakes to modulate subcutaneous fat mass (Table 6). Saturated, monounsaturated and polyunsaturated fatty acid intake did not interact with GRS to influence subcutaneous fat mass. In addition to nutrient intake alcohol and coffee intake did not have an interaction with GRS to affect subcutaneous fat (Table 6). However, physical activity had an interaction with GRS to modulate subcutaneous fat (*P* = 0.002). In low physical activity, subjects with the high-GRS had an increase of subcutaneous fat by 2.589 compared to the low-GRS (*P* < 0.001; Table 6). However, subcutaneous fat mass had no significant association with GRS in high physical activity. Figure 1 showed that subcutaneous fat amount was much higher in the persons having low physical activity than those with high physical activity (*P* < 0.001). Moreover, subcutaneous fat amount was higher in the carriers with high-GRS than those with low-GRS only in low physical activity.

**Table 6:** Interaction of dietary and lifestyle factors and genetic risk scores (GRS) of model 7^1^ in the risk of subcutaneous fat contents

	Low- GRS^2^ (*n* = 999)	Medium-GRS (*n* = 1551)	High-GRS (*n* = 744)	Gene-lifestyles interaction *P* value^3^
	OR	OR (CI)	OR (CI)	
Low energy	1	1.757(1.294~2.387)^***^	2.515(1.762~3.590)^***^	0.508
High energy^4^		1.676(1.129~2.487)^*^	1.842(1.123~3.021)^*^	
Low carbohydrate	1	1.570(0.898~2.745)	2.019(1.034~3.944)^*^	0.992
High carbohydrate^5^		1.732(1.323~2.268)^***^	2.264(1.645~3.116)^***^	
Low protein	1	1.890(1.273~2.806)^**^	2.284(1.428~3.655)^**^	0.628
High protein^6^		1.578(1.160~2.147)^**^	2.189(1.516~3.159)^***^	
Low fat	1	1.728(1.260~2.371)^**^	2.326(1.599~3.384)^***^	0.996
High fat^7^		1.630(1.116~2.381)^*^	2.060(1.307~3.245)^**^	
Low SFA	1	1.875(1.350~2.604)^***^	2.333(1.590~3.424)^***^	0.725
High SFA^8^		1.494(1.044~2.139)^*^	2.123(1.370~3.289)^**^	
Low MUFA	1	1.764(1.276~2.439)^**^	2.465(1.689~3.597)^***^	0.940
High MUFA^9^		1.599(1.106~2.312)^*^	1.913(1.223~2.991)^**^	
Low PUFA	1	1.556(1.123~2.157)^**^	1.978(1.336~2.927)^**^	0.495
High PUFA^10^		1.789(1.243~2.574)^**^	2.403(1.563~3.693)^***^	
Low alcohol drinking	1	1.743(1.347~2.256)^***^	2.232(1.644~3.030)^***^	0.804
High alcohol drinking^11^		1.519(0.751~3.073)	2.444(1.018~5.865)^*^	
Low coffee intake	1	1.809(1.255~2.607)^**^	2.697(1.734~4.195)^***^	0.761
High coffee intake^12^		1.633(1.181~2.257)^**^	1.936(1.325~2.829)^**^	
Low physical activity	1	2.113(1.593~2.804)^***^	2.589(1.855~3.615)^***^	0.002
High physical activity^13^		0.937(0.594~1.478)	1.452(0.831~2.540)	

*SFA* saturated fatty acid, *MUFA* monounsaturated fatty acid, *PUFA* polyunsaturated fatty acids

Values represent odd ratios (OR) and 95% confidence intervals (CI)

^1^GRS was divided into 3 categories by tertiles of GRS in the model which included *IL17A* rs4711998, *ADCY2* rs326149, *ESRRG* rs4846514, *CYFIP2* rs733730, *TCF7L2* rs7917983, *ZNF766* rs41497444 and *TGFBR3* rs7526590

^2^Reference was the Low-GRS

^3^Multivariate regression models include the corresponding main effects, interaction terms of gene and main effects (lifestyles including nutrient intake), and potential confounders such as age, gender, BMI, residence area, waist circumference, hip circumference, body fat, alcohol intake, physical activity, coffee intake, smoking, and energy intake

The cutoff points were assigned by 75 percentiles of each parameters for the high group and they were as following: 100% estimated energy intake^4^, 65% carbohydrate (CHO) intake^5^, 13% protein intake^6^, 15% fat intake^7^, 2.8% saturated fatty acids (SFA)^8^, 3.7% monounsaturated fatty acids (MUFA)^9^, 2.1% polyunsaturated fatty acids (PUFA)^10^, 20 g alcohol per day^11^, 10 cups of coffee per week^12^, and 1 h moderate physical activity per day^13^

^*^Significantly different from Low-GRS in logistic regression analysis at ^*^ *P* < 0.05, ^**^ *P* < 0.01, ^***^ *P* < 0.001

## Discussion

This study evaluated the effects of a high genetic risk score (GRS) for subcutaneous fat on fat partitioning and on the risk of metabolic disease in a human population, as well as the effect of visceral vs subcutaneous fat themselves on metabolic disease. Our results confirmed that having a high GRS more than doubled the likelihood of having a greater subcutaneous fat mass. We also confirmed that high visceral fat mass, not subcutaneous fat mass, was a risk factor for metabolic syndrome. However, a high GRS for subcutaneous fat could not be shown to protect against metabolic disease even though it increased subcutaneous fat. This may be involved in the selection of subjects in studies with GRS. We excluded subjects with low visceral fat mass when adjusting form BMI in Models 1 & 2, since they exhibited a big difference in total fat and BMI between the high and low subcutaneous fat groups. GWAS and genetic variant related studies were conducted with HS-HV and LS-HV groups which had similar total fat mass.

It is well established that the location of fat deposits in the body partially determines their physiological impact in humans [1, 2]. It has been suggested that visceral fat should be considered “bad fat” because it increases the risk of metabolic diseases, and that subcutaneous fat should be considered “good fat” because of its intrinsic metabolic benefits [20]. This notion received some support from a mouse study in which either subcutaneous or visceral fat from donor mice were transplanted into either peripheral or intra-abdominal regions of recipient mice [21]. Their study revealed little effect of transplanting visceral fat into either location, but beneficial effects on insulin sensitivity when transplanting subcutaneous fat into either peripheral or especially intra-abdominal regions. Although the results may not be directly applicable to humans, they do suggest that visceral and subcutaneous fat are different in their effects and that the benefits of subcutaneous fat benefits may be linked to properties of the fat itself and not just the location. However, it remains unknown how much of the benefit of subcutaneous fat is due to the fact that it is not deposited as visceral fat since the portioning of excess energy as fat into one compartment would decrease its accumulation in the other compartment. In this study, subcutaneous fat itself did not appear to be beneficial because the metabolic indices of dyslipidemia in LS-LV group was the same or lower than the HS-LV group, suggesting that having low amounts of both visceral and subcutaneous fat is ideal.

Because of the negative health consequences of excessive visceral fat, many studies have identified risk factors for visceral fat accumulation. It is known that advancing age increases the accumulation of visceral fat [22], as does the consumption of fructose sugar [23] and a sedentary lifestyle [24]. In the present study, as expected, people with high visceral fat were older, and had higher energy intakes, but fructose consumption was not able to be identified. Activity levels were the only significant gene-lifestyle interaction shown in this study. High physical activity was associated with less likelihood of being in the high subcutaneous fat group; although, when looking at the population as a whole, there were substantially more subjects at the highest level of exercise in the LS groups. This apparent inconsistency may be due to exercise lowering the amount of both fats, but having the most profound effect on visceral fat. In the population as a whole, subjects with high visceral fat content had higher energy, fat and protein intakes. Subjects with high subcutaneous fat had lower carbohydrate intakes and better insulin sensitivity, which may be due to consuming less fructose, since fructose consumption has been shown to favor partitioning of fat to the visceral region and is associated with greater insulin resistance as HOMA-IR [25].

The reasons for the different effects between visceral and subcutaneous adipose tissue are not fully understood. However, it may in part be due to a lack of self-limiting feedback from visceral fat that would prevent it’s unconstrained expansion. Adiponectin and leptin secretions are both involved in regulating body fat and metabolism. However, leptin production is primarily from subcutaneous fat and not from visceral adipose tissue [26]. Likewise, increasing BMI decreases overall adiponectin secretion, but is tissue specific with decreases in visceral adipose tissue, but not in subcutaneous adipose tissue [4]. Therefore, production of these two important adipokines that can both limit its expansion and prevent fat-induced dyslipidemia appears to be tissue-specifically limited in visceral adipose tissue, but not subcutaneous adipose tissue.

This study has important limitations. It is cross-sectional in nature, so cause and effect relationships cannot be evaluated. Skin fold measurement for determining subcutaneous fat mass is highly precise although it is commonly used in large-scale studies [11]. In this study, skilled technicians measured it with good quality control and it was validated to be reliable. Additionally, the complexity of genetic and lifestyle interactions with fat partitioning is not fully understood and some important factors may not have been adequately controlled in the GRS models. Furthermore, fructose consumption is known to be an important contributor to visceral fat accumulation, but the data did not specifically address dietary fructose, just carbohydrate as a whole.

## Conclusion

We demonstrated that high visceral fat led to dyslipidemia and blood glucose dysregulation in the Korean adult population in this study. Although the adverse metabolic effects of visceral fat were more pronounced than with subcutaneous fat and partitioning to the subcutaneous compartment would be beneficial, the subjects with low visceral and subcutaneous fat exhibited the best metabolic profiles. The major novel findings of this study were that a GRS for higher subcutaneous fat deposition was composed of genetic variants influencing proinflammatory cytokine signaling, and the GRS can be a major genetic predictor of subcutaneous fat in the Korean population, and possibly Asian populations. Subcutaneous fat contents had an interaction only with physical activity and the genetic impact can be avoided by high physical activity if carriers with high-GRS are wanted.

## Data Availability

The datasets used during the present study are available from the corresponding author upon reasonable request.

## Abbreviations

ADCY2: Adenylate cyclase 2
ALDH2: Aldehyde dehydrogenase 2 family member
ANOVA: Analysis of variance
BMI: Body mass index
CI: Confidence intervals
CNTN4: Contactin 4
CPEB4: Cytoplasmic polyadenylation element binding protein 4
CRP: C-reactive protein
CVC: Cross-validation consistency
CYFIP2: Cytoplasmic FMR1 interacting protein 2
ESRRG: Estrogen related receptor gamma
GLM: General linear model
GMDR: Generalized multifactor dimensionality reduction
GRS: Genetic risk scores
GWAS: Genome wide association study
HDL: High-density lipoprotein
HOXC13: Homeobox C13
HS-HV: High subcutaneous fat and high visceral fat
HS-LV: High subcutaneous fat and low visceral fat
IL17A: Interleukin 17A
IL5RA: Interleukin 5 receptor subunit alpha
IL-6: Interleukin-6
KoGES: Korean Genome and Epidemiology Study
LS-HV: Low subcutaneous fat and high visceral fat
LS-LV: Low subcutaneous fat and low visceral fat
LYPLAL1: Lysophospholipase like 1
ORs: Odds ratios
RSPO3: R-spondin 3
SNP: Single-nucleotide polymorphisms
SQFFQ: Semi-quantitative food frequency questionnaires
TBX15: T-box 15
TCF7L2: Transcription factor 7 like 2
TEBA: Test balance accuracy
TGFBR3: Transforming growth factor beta receptor 3
TNF- α: Tumor necrosis factor-α
TRBA: Trained balance accuracy
VLDL: Very low-density lipoprotein
ZNF766: Zinc finger protein 766

## References

[1] Seo MH, Lee W-Y, Kim SS, Kang J-H, Kang J-H, Kim KK (2019). 2018 Korean Society for the Study of obesity guideline for the Management of Obesity in Korea. J Obes Metab Syndr.

[2] Hamdy O, Porramatikul S, Al-Ozairi E (2006). Metabolic obesity: the paradox between visceral and subcutaneous fat. Curr Diabetes Rev.

[3] Medina-Urrutia A, Posadas-Romero C, Posadas-Sanchez R, Jorge-Galarza E, Villarreal-Molina T, Gonzalez-Salazar Mdel C (2015). Role of adiponectin and free fatty acids on the association between abdominal visceral fat and insulin resistance. Cardiovasc Diabetol.

[4] Sato F, Maeda N, Yamada T, Namazui H, Fukuda S, Natsukawa T (2018). Association of Epicardial, visceral, and subcutaneous fat with Cardiometabolic diseases. Circ J.

[5] Ryden M, Arner P (2017). Subcutaneous adipocyte lipolysis contributes to circulating lipid levels. Arterioscler Thromb Vasc Biol.

[6] Schleinitz D, Bottcher Y, Bluher M, Kovacs P (2014). The genetics of fat distribution. Diabetologia..

[7] Adamska-Patruno E, Goscik J, Czajkowski P, Maliszewska K, Ciborowski M, Golonko A, et al. The MC4R genetic variants are associated with lower visceral fat accumulation and higher postprandial relative increase in carbohydrate utilization in humans. Eur J Nutr. 2019. 10.1007/s00394-019-01955-0 PMC6768895 30945034

[8] Park S, Daily JW, Zhang X, Jin HS, Lee HJ, Lee YH (2016). Interactions with the MC4R rs17782313 variant, mental stress and energy intake and the risk of obesity in Genome Epidemiology Study. Nutr Metab (Lond).

[9] Wang T, Ma X, Peng D, Zhang R, Sun X, Chen M (2016). Effects of Obesity Related Genetic Variations on Visceral and Subcutaneous Fat Distribution in a Chinese Population. Sci Rep.

[10] Hong KW, Kim SH, Zhang X, Park S (2018). Interactions among the variants of insulin-related genes and nutrients increase the risk of type 2 diabetes. Nutr Res.

[11] Rönnecke E, Vogel M, Bussler S, Grafe N, Jurkutat A, Schlingmann M (2019). Age- and sex-related percentiles of skinfold thickness, waist and hip circumference, waist-to-hip ratio and waist-to-height ratio: results from a population-based pediatric cohort in Germany (LIFE child). Obes Facts.

[12] Madden AM, Smith S (2016). Body composition and morphological assessment of nutritional status in adults: a review of anthropometric variables. J Hum Nutr Diet.

[13] Chen CH, Chen YY, Chuang CL, Chiang LM, Chiao SM, Hsieh KC (2014). The study of anthropometric estimates in the visceral fat of healthy individuals. Nutr J.

[14] Ping Z, Pei X, Xia P, Chen Y, Guo R, Hu C (2018). Anthropometric indices as surrogates for estimating abdominal visceral and subcutaneous adipose tissue: a meta-analysis with 16,129 participants. Diabetes Res Clin Pract.

[15] Kim Da Sol, Kim Byoung Chul, Daily James W., Park Sunmin (2017). High genetic risk scores for impaired insulin secretory capacity doubles the risk for type 2 diabetes in Asians and is exacerbated by Western-type diets. Diabetes/Metabolism Research and Reviews.

[16] Ahn Y, Kwon E, Shim JE, Park MK, Joo Y, Kimm K (2007). Validation and reproducibility of food frequency questionnaire for Korean genome epidemiologic study. Eur J Clin Nutr.

[17] Rabbee N, Speed TP (2006). A genotype calling algorithm for affymetrix SNP arrays. Bioinformatics..

[18] Chen GB, Liu N, Klimentidis YC, Zhu X, Zhi D, Wang X (2014). A unified GMDR method for detecting gene-gene interactions in family and unrelated samples with application to nicotine dependence. Hum Genet.

[19] Uma Jyothi K, Reddy BM (2015). Gene-gene and gene-environment interactions in the etiology of type 2 diabetes mellitus in the population of Hyderabad. India Meta Gene.

[20] Taylor G (2007). Science to Practice: good fat, bad fat--does location matter?. Radiology.

[21] Tran TT, Yamamoto Y, Gesta S, Kahn CR (2008). Beneficial effects of subcutaneous fat transplantation on metabolism. Cell Metab.

[22] Al-Sofiani ME, Ganji SS, Kalyani RR (2019). Body composition changes in diabetes and aging. J Diabetes Complicat.

[23] Rosset R, Surowska A, Tappy L (2016). Pathogenesis of cardiovascuar and metabolic diseases: are fructose-containing sugars more involved than other dietary calories?. Curr Hypertens Rep.

[24] Goedecke JH, Micklesfield LK (2014). The effect of exercise on obesity, body fat distribution and risk for type 2 diabetes. Med Sport Sci.

[25] Stanhope KL, Schwarz JM, Keim NL, Griffen SC, Bremer AA, Graham JL (2009). Consuming fructose-sweetened, not glucose-sweetened, beverages increases visceral adiposity and lipids and decreases insulin sensitivity in overweight/obese humans. J Clin Invest.

[26] Banerji Mary Ann, Faridi Nuzhat, Atluri Rajesh, Chaiken Rochelle L., Lebovitz Harold E. (1999). Body Composition, Visceral Fat, Leptin, and Insulin Resistance in Asian Indian Men1. The Journal of Clinical Endocrinology & Metabolism.

